# Dietary Supplementation with 23-Hydroxy Ursolic Acid Reduces the Severity and Incidence of Acute Experimental Autoimmune Encephalomyelitis (EAE) in a Murine Model of Multiple Sclerosis

**DOI:** 10.3390/nu16030348

**Published:** 2024-01-25

**Authors:** Reto Asmis, Megan T. Medrano, Carol Chase Huizar, Wendell P. Griffith, Thomas G. Forsthuber

**Affiliations:** 1 Department of Internal Medicine, Wake Forest School of Medicine, Winston-Salem, NC 27157, USA; 2 Department of Molecular Microbiology and Immunology, University of Texas at San Antonio, San Antonio, TX 78249, USA; megan.medrano@utsa.edu (M.T.M.);; 3 Department of Chemistry, University of Texas at San Antonio, San Antonio, TX 78249, USA

**Keywords:** inflammation, redox biology, phytochemical, disease prevention, multiple sclerosis

## Abstract

23-Hydroxy ursolic acid (23-OH UA) is a potent atheroprotective and anti-obesogenic phytochemical, with anti-inflammatory and inflammation-resolving properties. In this study, we examined whether dietary 23-OH UA protects mice against the acute onset and progression of experimental autoimmune encephalomyelitis (EAE), a mouse model of multiple sclerosis (MS). Female C57BL/6 mice were fed either a defined low-calorie maintenance diet (MD) or an MD supplemented with 0.2% wgt/wgt 23-OH UA for 5 weeks prior to actively inducing EAE and during the 30 days post-immunization. We observed no difference in the onset of EAE between the groups of mice, but ataxia and EAE disease severity were suppressed by 52% and 48%, respectively, and disease incidence was reduced by over 49% in mice that received 23-OH UA in their diet. Furthermore, disease-associated weight loss was strikingly ameliorated in 23-OH UA-fed mice. ELISPOT analysis showed no significant differences in frequencies of T cells producing IL-17 or IFN-γ between 23-OH UA-fed mice and control mice, suggesting that 23-OH UA does not appear to regulate peripheral T cell responses. In summary, our findings in EAE mice strongly suggest that dietary 23-OH UA may represent an effective oral adjunct therapy for the prevention and treatment of relapsing–remitting MS.

## 1. Introduction

Multiple sclerosis (MS) is a chronic inflammatory and neurodegenerative disease of the CNS, characterized by inflammatory demyelination, axonal damage, and neuron loss [1]. In 2020, an estimated 2.8 million people lived with MS worldwide, over 400,000 in the U.S. alone. Approximately 75% of the people affected were women. MS prevalence has increased in every world region since 2013 [2], though the reasons for this are not fully understood. MS susceptibility is thought to be promoted by both genetic and environmental factors. The MHC II allele HLA-DR2b (DRB1*1501) is the most common genetic link to MS, although the mechanisms by which HLA-DR2b may promote disease progression are not fully understood [3,4,5].

MS lesions can appear throughout the CNS, which is why this demyelinating disease presents with a range of syndromes, including limb weakness and sensory loss, ataxia caused by cerebellar lesions, monocular vision loss due to optic neuritis, and/or double vision caused by brainstem dysfunction [6]. While approximately 10–15% of patients show a progressive clinical course of the disease from the onset, approximately 85% of patients show the relapsing–remitting form of MS (RRMS), and secondary progressive MS (SPMS) typically develops in many of these patients after 10 to 20 years, ultimately resulting in impaired mobility and cognition [7]. On average, patients with MS have life expectancies that are shortened by 7–14 years [8,9].

MS is thought to be an autoimmune-mediated central nervous system (CNS) disorder where neuroantigen-reactive T cells, B cells, as well as cells of the innate immune system promote disease pathology [10,11,12,13,14,15,16,17]. Data from the experimental autoimmune encephalomyelitis (EAE) animal models of MS show that myelin-specific T cells promote disease pathology by releasing proinflammatory cytokines, such as IL-17, IFN-γ, TNF, and GM-CSF, which trigger demyelination and axonal transection [18,19].

The histopathological hallmarks of MS, i.e., inflammation, demyelination, neurodegeneration, and glial scar formation are present in all forms of MS, but there are significant differences between RRMS and SPMS, whereas SPMS and PPMS overlap [20]. In RRMS, active or chronic-active lesions are frequently present, whereas inactive lesions are typical for PPMS and SPMS, which are dominated by innate immune and glial cells [17,21].

A growing number of disease-modifying therapies are available that can reduce morbidity, especially when started early in the course of the disease [22]. However, the etiology of MS remains incompletely understood and, to this day, there is no cure for MS, nor effective treatments for progressive forms of the disease. Thus, there is a compelling need for novel therapeutics that target mechanisms underlying progressive MS, the neurodegenerative components of this disease, as well as CNS repair mechanisms [1].

Of the three established animal models used by the scientific community to study MS—(a) viral models, including Theiler’s murine encephalomyelitis (TMEV), (b) disease models that are induced by toxic chemicals such as cuprizone, and (c) experimental autoimmune encephalomyelitis (EAE) and variations thereof [23,24]—each model mimics similar features of MS, but the underlying molecular mechanism and pathological features vary dramatically. The most common animal model used is the EAE model, which is particularly useful for the study of neuroinflammatory pathways and is commonly used as a “proof-of-principle” model to test novel drugs and treatments [25].

23-Hydroxy ursolic acid (23-OH UA) is a naturally occurring triterpenoid found in the leaves of *Lagerstroemia speciosa*, or giant crepe myrtle, native to South East Asia and the leaves and twigs of *Juglans sinensis*, a walnut tree found in East Asia [26]. 23-OH UA is structurally closely related to ursolic acid (UA), a well-studied natural compound with numerous purported health benefits found in herbs and the peels of fruit. In an established mouse model of human atherosclerosis, we showed that both dietary 23-OH UA and UA have potent atheroprotective and anti-obesogenic activity in mice, and they protect blood monocytes against nutrient-stress-induced dysfunction [27]. Despite similar mechanisms of action, dietary 23-OH UA is significantly more effective in preventing atherosclerotic lesion formation than UA. In a recent follow-up study using a mouse model of diet-induced obesity, we confirmed the potent anti-obesogenic properties of 23-OH UA and demonstrated that 23-OH UA also improves glucose tolerance, prevents hyperleptinemia, restores blood monocyte function, and suppresses the recruitment of monocyte-derived macrophages into adipose tissues in these mice. The mechanisms underlying the benefits of 23-OH UA appear to involve the conversion or “reprogramming” of macrophages into a unique transcriptionally hyperactive phenotype. Based on targeted gene profiling data, this macrophage phenotype appears to be highly resistant to oxidative stress, equipped with high metabolic and fuel flexibility, and has potent anti-inflammatory activity [27]. Inflammatory macrophages, dendritic cells, and microglia critically contribute to the induction and propagation of neuroinflammation. Therefore, we examined in this study whether dietary supplementation with 23-OH UA exerts similar anti-inflammatory effects on CNS innate immune cells and whether 23-OH UA protects mice against experimental autoimmune encephalomyelitis (EAE) in a mouse model of human MS [25].

## 2. Materials and Methods

### 2.1. Animals

Female C57BL/6J (stock number 000664) were obtained from Jackson Laboratory. All mice were maintained in colony cages on a 12 h light/12 h dark cycle and fed a normal mouse laboratory diet unless otherwise stated. Twenty 6-week-old female C57BL/6 mice were randomized into two groups of ten mice. One group was fed a defined low-calorie maintenance diet (MD, AIN-93G, BioServ, Flemington, NJ, USA), and the other group was fed an MD supplemented by BioServ with 0.2% wgt/wgt 23-OH UA for 5 weeks prior to immunization and throughout the 30-day post-immunization period. 23-OH UA was custom synthesized by WuXi App Tec (Shanghai, China) from Asiatic acid according to a 6-step strategy developed by Bore et al. [26]. The structure was confirmed by MS and NMR. The purity of the final product was determined by HPLC and LCMS and was >99%. The pure compound was sent to Bioserv, where it was added to the AIN-93G powder prior to generating pellets. Diets and water were provided ad lib. All experiments were conducted in accordance with animal protocols approved by the University of Texas at San Antonio Institutional Animal Care and Use Committee.

### 2.2. EAE Induction

After 5 weeks (day 0) on MD or MD supplemented with 23-OH UA, EAE was actively induced by subcutaneous injection with 100 mg of mouse MOG_35–55_ emulsified in 50 µL of complete Freund’s adjuvant (CFA) containing 5 mg/mL *Mycobacterium tuberculosis* H37Ra, as described previously [28]. A total of 200 ng of *Bordetella pertussis* toxin was administered intraperitoneally (i.p.) on day 0 and day 2 after immunization. Mice were weighed and clinically scored daily for 30 days. On day 30, splenocytes were isolated and plated on ELISPOT plates that were pre-coated with anti-IL-17 or anti-IFN-g antibodies, restimulated with MOG_35–55_, and subsequently stained and analyzed using a Series 2 ImmunoSpot analyzer (Shaker Heights, Cleveland, OH, USA).

### 2.3. EAE Disease and Ataxia Scoring

EAE disease was scored as previously described on a scale of 0–5 (0: no clinical disease; 1: flaccid tail; 2: partial hind-limb paralysis; 3: total hind-limb paralysis; 4: front and hind-limb paralysis; 5: moribund or dead) [28]. Ataxia was assessed and scored as described on a scale of 0–3 using the following four tests: ledge test (0: balanced and graceful; 1: loses footing but still shows coordination; 2: cannot use hind legs well, slipping but balanced; 3: legs shaking significantly, not graceful), hindlimb clasping (0: hind legs move outward; 1: 50% of the 10 s one hind limb is moved inward; 2: 50% of the 10 s both hind limbs are moved inward; 3: >50% of the 10 s both hind limbs are near the abdomen), gait (0: normal movement; 1: slight tremor or waddle during movement; 2: hind legs are shaking during movement, severe tremor; 3: hind legs are dragging and abdomen near the floor), and kyphosis (0: flat back; 1: initial curvature of back but able to flatten while moving; 2: consistent mild curvature of the back; 3: consistent severe curvature of the back) [28]. The ataxia score was calculated as the sum of scores from all four tests. Four mice died in the control group, and three died in the group of mice fed a 23-OH UA-supplemented diet. All mice died between days 13 and 16. To prevent data bias, dead mice were assigned the maximum score in each category and a 30% weight loss for the remaining days of the study.

### 2.4. Cytokine ELISPOT Assay

Cytokine ELISPOT assay was performed and spots were analyzed as described previously [18,19]. In brief, ELISPOT plates (Millipore, Billerica, MA, USA) were precoated with anti-mouse-IFN-γ mAb (eBioscience, San Diego, CA, USA, AN-18) and anti-mouse-IL-17 mAb (17F3, Bio X Cell, Lebanon, NH, USA). Splenocytes (5 × 10^5^ cells/well) were restimulated with MOG35–55 peptide in HL-1 medium (Lonza, Basel, Switzaerland) at 37 °C, 5% CO_2_ for 24 h. Biotinylated anti-mouse-IFN-γ mAb (R4-6A2, eBioscience/Thermo Fisher, Waltham, MA, USA) and anti-mouse-IL-17 mAb (TC11-8H4, BioLegend, San Diego, CA, USA) were then added overnight at 4 °C, followed by incubation with streptavidin alkaline phosphatase (Invitrogen, Waltham, MA, USA) for 2 h at room temperature and developing with BCIP/NBT Phosphatase Substrate (KPL, Gaithersburg, MD, USA). After plate development, image analysis of spots was performed on a Series 2 ImmunoSpot analyzer (Cellular Technology Limited, Cleveland, OH, USA). Results for antigen-specific spot-forming cells were normalized with a negative control containing peptide-free media. All measurements were performed in triplicate.

### 2.5. Liquid Chromatography–Mass Spectrometry

23-OH UA and glycyrrhetinic acid (GA, the internal standard) stock solutions were prepared in methanol to give a final concentration of 1.0 mg/mL. From these, 2 ppm working stock solutions were prepared by dilution in methanol. The GA working solution (10 ppm) was prepared in methanol from the 1.0 mg/mL stock solution. All solutions were kept at 4 °C and brought to room temperature prior to use.

The volume of commercial plasma (P9275, Sigma-Aldrich, St. Louis, MO, USA) required for the analysis was spiked with GA to a final concentration of 100 ppb. Calibration standard solutions with concentrations of 0, 10, 20, 50, 100, 200, 350, and 500 ppm were prepared by spiking the required volume of the 2 ppm 23-OH UA stock solution into 50 mL volumes of the plasma containing 100 ppb GA. For samples, 50 mL of plasma was mixed with 50 mL of plasma containing 100 ppb GA. To all calibration standards and samples, 0.5 mL of ethyl acetate was added, vortexed for approximately 10 s, allowed to sit for 5 min, and vortexed again for 10 s. This was followed by centrifugation for 10 min at 12,000 rpm. An amount of 400 mL of the ethyl acetate layer was carefully pipetted into clean microcentrifuge tubes and evaporated to dryness in a Speedvac. Calibration standards and samples were reconstituted in 50 mL of methanol and transferred into injection vials for LC-MS/MS analysis.

Chromatographic separations and mass spectrometry analyses were performed on an Acquity UPLC system directly coupled to an Acquity triple quadrupole detector (Waters Corp., Milford, MA, USA). For chromatography, isocratic elution was performed using a mixture of 80% acetonitrile and 20% water containing 10 mM ammonium formate. Separation was achieved at a constant flow rate of 0.15 mL/min using a BDS Hypersil C18 column (50 × 2.1 mm, 5 μm particle size) from Thermo Electron Corporation (Waltham, MA, USA) with a constant column temperature of 40 °C. For each analysis, 5 μL of the sample was injected into the column. Mass spectrometry analysis was conducted in negative ionization mode using multiple reaction monitoring (MRM) with capillary, cone, and extraction potentials maintained at 1.5 kV, 80 V, and 3 V, respectively. The source block temperature was 110 °C and the desolvation temperature was 350 °C with a desolvation gas flow of 600 L/h. The cone gas flow rate was 10 L/h. Quantitation of 23-OH UA was conducted in multiple reaction monitoring (MRM) mode using the transition *m*/*z* 471.40/471.30 to monitor the [M–H^+^]^−^. The transition *m*/*z* 469.3/469.2 was used to monitor the internal standard GA. Collision energy (CE) was set at 20 eV. Analyses of standards and samples were conducted in triplicate, and peak areas were used for quantitation. MassLynx v.4.1 software was used for data acquisition and analysis (Waters Corp., Milford, MA, USA).

### 2.6. Statistical Analyses

Mean values between the two experimental groups were compared by an unpaired two-tailed Student *t*-test (SigmaPlot 15). Normality was tested with the Shapiro–Wilk test. Disease onset was determined for both the EAE disease score and the combined Ataxia scores using one-way ANOVA on repeated measures and the Holm–Sidak test to determine on which day mean scores were significantly elevated (*p* > 0.05), setting values at day 0 as the control group. Unless stated otherwise, data are expressed as mean ± standard error of the mean (SEM). *p* < 0.05 was set as the statistical significance level.

## 3. Results

### 3.1. Dietary 23-OH UA Prevents Weight Loss Associated with EAE

**Figure 1:**
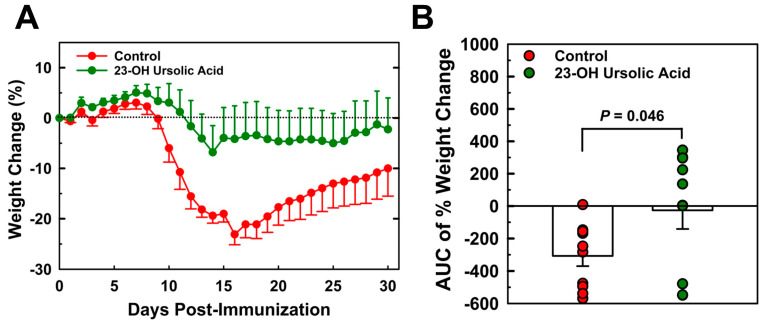
Dietary 23-OH UA prevents weight loss in EAE mice. Female C57BL/6 mice were maintained on either an MD (●) or an MD supplemented with 23-OH UA (●) before EAE was actively induced by subcutaneous injection with mouse MOG_35–55_ emulsified in complete Freund’s adjuvant and for 30 days after immunization. Weights were recorded daily for 30 days post-immunization (**A**) and changes in body weight were calculated as AUC (**B**). Results are expressed as means +/− SE (n = 10 per group).

**Figure 2:**
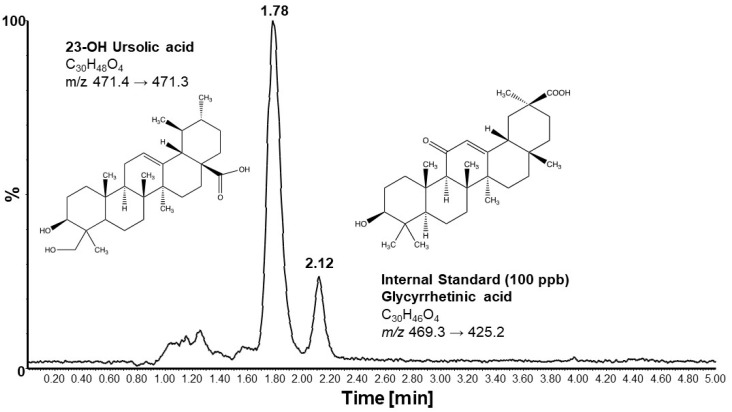
Representative chromatogram of LCMS an analysis of mouse plasma. For details, see Section 2.

Mice maintained on an MD and immunized with MOG_35–55_ began losing weight on day 9 post-immunization (Figure 1A). Weight loss peaked on day 16, on average at 23% of the original weight, before slowly recovering. By day 30, the mean body weight was still 10% below the original weight. Mice that received 23-OH UA in their diets were, on average, able to maintain their body weight despite the immunization (Figure 1B). Plasma levels of 23-OH UA were assessed by LCMS (Figure 2). The mean plasma concentration of 23-OH UA reached 7.4 ± 4.7 mM by day 30. As expected, no 23-OH UA was detected in the plasma samples from mice that received MD without 23-OH UA supplementation.

### 3.2. Dietary 23-OH UA Reduces EAE Disease and Ataxia Severity

**Figure 3:**
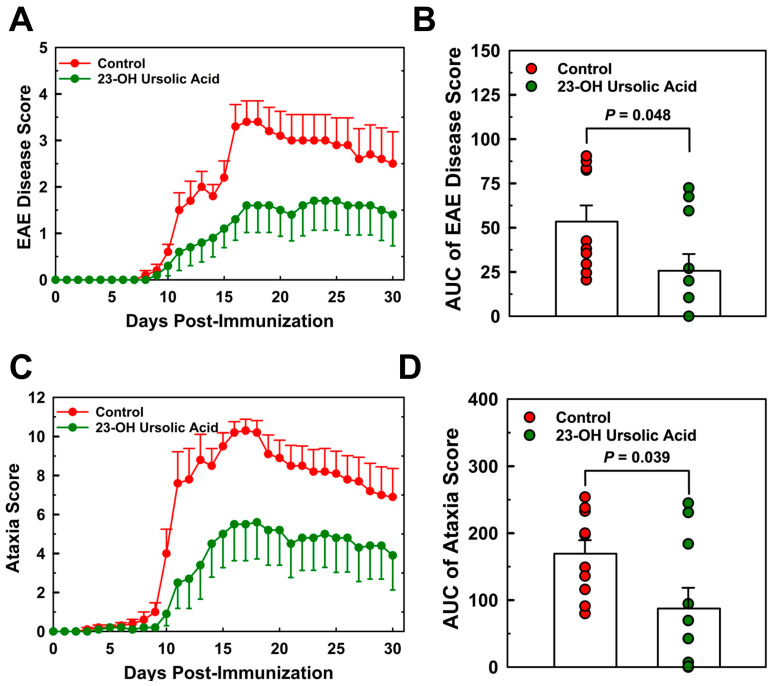
Dietary 23-OH UA reduces disease severity in EAE mice. EAE was actively induced in female C57BL/6 mice maintained on either an MD (●) or an MD supplemented with 23-OH UA (●), as described in Figure 1. Clinical disease course was monitored daily and EAE (**A**) and ataxia severity (**C**) were scored for 30 days post-immunization, as described under “Methods”. EAE disease and ataxia severity (**B**) were calculated as AUC ((**B**) + (**D**)). Results are expressed as means +/− SE (n = 10 per group).

MOG_35–55_ immunized EAE mice maintained on an MD began showing signs of symptoms around day 8 post-immunization (Figure 3A). Disease severity increased rapidly thereafter and peaked on day 17 at a mean score of 3.4. By day 30, the mean EAE disease score had decreased to 2.6. The rapid increase in the EAE disease score was mirrored by the combined ataxia score, although the onset of visual physical impairment based on the criteria for the ataxia score was already observed around day 8 post-immunization (Figure 3C).

**Figure 4:**
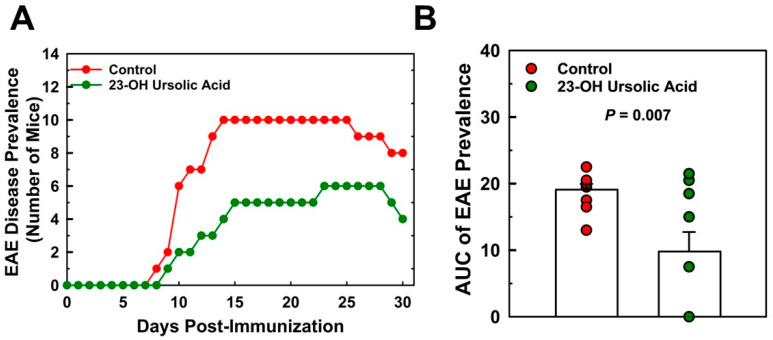
Dietary 23-OH UA reduces disease prevalence in EAE mice. EAE was actively induced in female C57BL/6 mice maintained on either an MD (●) or an MD supplemented with 23-OH UA (●), as described in Figure 1. EAE disease incidence (**A**) was monitored daily for 30 days post-immunization, as described under Section 2. Total EAE disease prevalence was calculated as the area under the curve (AUC), i.e., the number of days a mouse scored at least a “1” on the EAE scoring scale for the 30-day period post-immunization (**B**). Results are expressed as means +/− SE (n = 10 per group).

Ataxia scores for the MD-fed control mice peaked at day 17 at a mean score of 10.3 and decreased to 7.0 at day 30. Feeding mice 23-OH UA protected mice from EAE, reduced EAE disease severity by 52% (Figure 3B), and dramatically improved the overall ataxia score by 48% (Figure 3D). The prevalence of EAE was reduced in 23-OH UA-fed mice, in line with the effect of 23-OH UA on disease severity (Figure 4A). The overall reduction in prevalence was 49% (Figure 4B). Of note, dietary 23-OH UA also delayed disease onset (Figure 3A,C). Initial increases in EAE disease scores were delayed by 5 days, from day 11 to day 16 post-immunization, whereas the first noted changes in ataxia were delayed by four days, from day 10 to day 14 post-immunization. While the reason for this delay is unclear at this time, one possibility is that 23-OH UA feeding may have affected the immunization of the mice.

### 3.3. Dietary 23-OH UA Does Not Affect Peripheral Neuroantigen-Specific T Cell Responses

**Figure 5:**
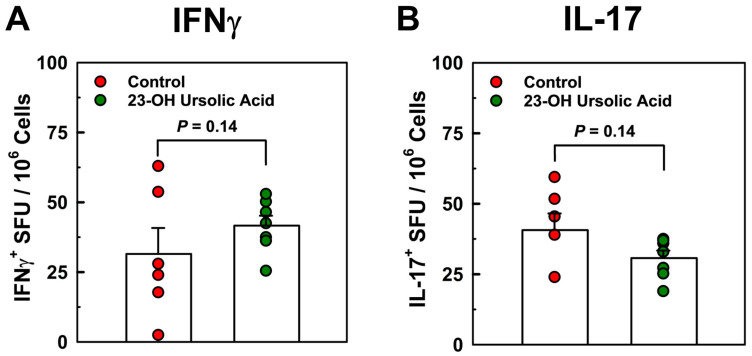
Protection conferred by dietary 23-OH UA is not associated with decreased frequency of Th1 (**A**) or Th17 cells (**B**)**.** Splenocytes were isolated from EAE mice maintained on either an MD (●) or an MD supplemented with 23-OH UA (●) on day 30 post-immunization, and Th17 or Th1 MOG_35–55_ peptide-specific T cell frequencies were measured using cytokine ELISPOT recall assays. Results are expressed as means +/− SE (controls: n = 6; 23-OH UA-treated: n = 7).

To address potential mechanisms underlying the potent protective effects of dietary 23-OH UA, we analyzed T cell responses in splenocytes in recall experiments with MOG_35–55_ peptides by cytokine ELISPOT assays. We observed no effect of dietary 23-OH UA on Th17 or Th1 antigen-specific T cell frequency in splenocytes isolated on day 30 post-immunization and recalled with MOG_35–55_ peptides (Figure 5), suggesting that 23-OH UA does not appear to target peripheral T cell responses.

## 4. Discussion

Here, we demonstrate that dietary supplementation with 23-OH UA potently protects mice against EAE in a preclinical model of human MS [25]. Ataxia and EAE disease severity and incidence were suppressed by approximately 50%, and EAE-associated weight loss was prevented. Surprisingly, dietary 23-OH UA did not appear to affect peripheral T cell responses in EAE mice, generally considered a myelin-specific T cell-mediated neuroinflammatory autoimmune disease [29,30,31]. Conceivably, the supplement may exert its effects on autoreactive T cells within the CNS, or, more likely, modulate the pathogenic functions of other cell types in the CNS. Along these lines, CNS-infiltrating dendritic cells or inflammatory macrophages may be the target of 23-OH UA during neuroinflammation and their T cell activating properties modulated, such as processing and presentation of neuroantigens, upregulation of MHC or costimulatory molecules, or cytokine production. Furthermore, the supplement may affect the expression of phagocytic receptors on CNS APCs. In addition, the migration of inflammatory cells, including encephalitogenic T cells and macrophages/dendritic cells, may be affected. In this vein, 23-OH UA may affect the migration of other immune cell populations to the CNS, for example, that of regulatory T cells (Treg). Increased migration of Tregs to areas of neuroinflammation may confer protection by downregulating the function of pathogenic T cells, infiltrating macrophages/dendritic cells, and/or CNS resident microglia.

We reported previously that dietary 23-OH UA protects mice against diet-induced obesity adipose tissue inflammation [32]. Of note, we monitored the mice for signs of toxicity of the supplement. We found no increase in liver toxicity as plasma AST and ALT activity levels remained unchanged [32]. We also found no changes in blood cell counts or any other adverse effects of 23-OH UA in either of our two published studies on 23-OH UA [27,32]. We went on to demonstrate that 23-OH UA exerts these anti-obesogenic properties through multiple mechanisms, which include improving glucose tolerance, reversing hyperleptinemia, and protecting blood monocytes against nutrient-stress-induced dysfunction [32]. By maintaining monocyte function in high-fat-diet-fed mice, 23-OH UA reduced the recruitment of monocyte-derived macrophage into the adipose tissue, thereby limiting adipose tissue inflammation. It is possible that dietary 23-OH UA has a similar effect in EAE mice, limiting the recruitment of monocyte-derived macrophages to sites of inflammation within the brain and restricting their disease contribution. MDM are considered the primary cell type involved in demyelination during EAE [33,34,35], suggesting that 23-OH UA’s inhibitory effects on MDM recruitment may both dampen neuroinflammation and prevent demyelination at sites of MDM accumulation.

A second mechanism underlying the anti-obesogenic and anti-inflammatory properties of 23-OH UA involves the reprogramming of macrophages by 23-OH UA into transcriptionally hyperactive phenotypes with anti-inflammatory and potentially inflammation-resolving properties [32]. Bone marrow-derived macrophages (BMDM) isolated from mice fed a high-calorie diet (HCD) supplemented with 23-OH UA showed a 39-fold increase in IL-10 mRNA expression. In addition to IL-10, we observed an increase in Nrf2 and Nrf2-dependent genes, including Gclc, Gclm, Gr, Grx1, Grx2, and Gsta3, involved in glutathione synthesis and homeostasis, as well as the antioxidant enzymes Cat, Gpx1, Gpx4, gpx7, and Prdx1, 3, 4, and 6, suggesting that this macrophage phenotype is well-protected against oxidative stress and that it may act as a sink for ROS and RNS in the microenvironment of sites of inflammation, thereby reducing oxidative damage.

Even more interesting was the finding that PPARγ-regulated genes were upregulated in these reprogrammed BMDM, including CD36, LPL, FABP4, and GLUT4 [32]. Zhang and colleagues report that ursolic acid, a structural analogue to 23-OH UA, also reduces the severity of EAE by acting as a PPARγ agonist, upregulating promyelinating factor CNTF in astrocytes and promoting oligodendrocyte maturation and remyelination [36]. Of note, astrocytes have been neglected in MS/EAE pathogenesis until recently [21,37,38]. Astrocytes were described in the earliest reports on MS and were even considered central to its pathogenesis by Charcot, who first established MS as a disease entity [21]. Accordingly, the activation and proliferation of microglia and astrocytes is observed in demyelinating lesions [37]. Moreover, the central role of astrocytes in maintaining the BBB is well established [21,37]. Nevertheless, until recently astrocytes were still considered passive bystanders of the autoimmune attack on the CNS by T cells and B cells [21,37,39]. Astrocytes can have both pathogenic and protective functions during neuroinflammation, can produce a plethora of pro- and anti-inflammatory mediators, and are intimately associated with the BBB and neurons as part of the “neuro-vascular unit”. Astrocytes are highly sensitive to changes in the CNS microenvironment, and they can adjust their functions depending on different stimuli [40]. Thus, astrocytes may be another plausible target for the therapeutic effects observed with 23-OH UA, which should be further explored in future studies.

In a mouse model of human atherogenesis, we showed that HCD supplementation with either ursolic acid or 23-OH UA inhibited dyslipidemia-induced monocyte priming and dysfunction, reduced weight gain, and protected these mice against atherosclerosis [27]. 23-OH UA, however, was significantly more potent than ursolic acid, possibly due to 23-OH UA’s greater predicted solubility. In this mouse model of atherosclerosis, both compounds appear to act through the same mechanisms, i.e., by preventing oxidation and the subsequent inactivation and degradation of MKP-1 [41,42], a master regulator of monocyte and macrophage function. Based on the molecular docking model provided by Zhang et al. showing ursolic acid binding to PPARγ [36], 23-OH UA should not only fit into the same binding pocket but would benefit from an additional hydrogen bridge with Glu291, suggesting that the protective properties of 23-OH UA in EAE, like ursolic acid, may also be mediated by PPARγ. The induction of PPARγ-dependent genes in BMDM isolated from C57BL/6 mice maintained on an HFD supplemented with 23-OH UA supports this hypothesis [32].

## 5. Conclusions

In summary, we provide evidence in a preclinical animal model of MS that 23-OH UA could be an effective oral supplement well suited as an adjunct therapy for reducing the development of acute episodes of relapsing–remitting MS and potentially slowing disease progression. Nevertheless, further studies are needed to determine the safety and efficacy of the supplement in MS patients. Furthermore, future studies should be aimed at providing a better mechanistic understanding of 23-OH UA function in the CNS during neuroinflammation, for example by examining effects on microglia, astrocytes, and the blood–brain barrier, which may affect the recruitment of inflammatory cells to the CNS. Moreover, the effect of the supplement on remyelination and oligodendrocytes/oligodendrocyte precursors should be further investigated. In this vein, 23-OH UA’s ability to potently induce Nrf2-dependent antioxidant systems and maintain redox homeostasis, combined with its ability to activate PPARγ-dependent signaling in immune cells—and very likely other tissues—may account for the broad range of health benefits reported for 23-OH UA and pave the way for improved myelin repair. Based on these findings in (T.G.F mice, we propose that 23-OH UA may be well suited as a dietary supplement for the prevention and management of MS.

## Data Availability

Data are contained within the article.

## References

[1] Correale J., Gaitan M.I., Ysrraelit M.C., Fiol M.P. (2017). Progressive multiple sclerosis: From pathogenic mechanisms to treatment. Brain.

[2] Walton C., King R., Rechtman L., Kaye W., Leray E., Marrie R.A., Robertson N., La Rocca N., Uitdehaag B., van der Mei I. (2020). Rising prevalence of multiple sclerosis worldwide: Insights from the Atlas of MS, third edition. Mult. Scler..

[3] Barcellos L.F., Sawcer S., Ramsay P.P., Baranzini S.E., Thomson G., Briggs F., Cree B.C., Begovich A.B., Villoslada P., Montalban X. (2006). Heterogeneity at the HLA-DRB1 locus and risk for multiple sclerosis. Hum. Mol. Genet..

[4] Patsopoulos N.A. (2018). Genetics of Multiple Sclerosis: An Overview and New Directions. Cold Spring Harb. Perspect. Med..

[5] Canto E., Oksenberg J.R. (2018). Multiple sclerosis genetics. Mult. Scler..

[6] Brownlee W.J., Hardy T.A., Fazekas F., Miller D.H. (2017). Diagnosis of multiple sclerosis: Progress and challenges. Lancet.

[7] Reich D.S., Lucchinetti C.F., Calabresi P.A. (2018). Multiple Sclerosis. N. Engl. J. Med..

[8] Kingwell E., van der Kop M., Zhao Y., Shirani A., Zhu F., Oger J., Tremlett H. (2012). Relative mortality and survival in multiple sclerosis: Findings from British Columbia, Canada. J. Neurol. Neurosurg. Psychiatry.

[9] Scalfari A., Knappertz V., Cutter G., Goodin D.S., Ashton R., Ebers G.C. (2013). Mortality in patients with multiple sclerosis. Neurology.

[10] Sospedra M., Martin R. (2005). Immunology of multiple sclerosis. Annu. Rev. Immunol..

[11] Huseby E.S., Liggitt D., Brabb T., Schnabel B., Ohlen C., Goverman J. (2001). A pathogenic role for myelin-specific CD8(+) T cells in a model for multiple sclerosis. J. Exp. Med..

[12] von Budingen H.C., Kuo T.C., Sirota M., van Belle C.J., Apeltsin L., Glanville J., Cree B.A., Gourraud P.A., Schwartzburg A., Huerta G. (2012). B cell exchange across the blood-brain barrier in multiple sclerosis. J. Clin. Investig..

[13] Krumbholz M., Derfuss T., Hohlfeld R., Meinl E. (2012). B cells and antibodies in multiple sclerosis pathogenesis and therapy. Nat. Rev. Neurol..

[14] Mann M.K., Ray A., Basu S., Karp C.L., Dittel B.N. (2012). Pathogenic and regulatory roles for B cells in experimental autoimmune encephalomyelitis. Autoimmunity.

[15] Steinman L., Martin R., Bernard C., Conlon P., Oksenberg J.R. (2002). Multiple Sclerosis: Deeper Understanding of Its Pathogenesis Reveals New Targets for Therapy. Annu. Rev. Neurosci..

[16] McFarland H.F., Martin R. (2007). Multiple sclerosis: A complicated picture of autoimmunity. Nat. Immunol..

[17] Charabati M., Wheeler M.A., Weiner H.L., Quintana F.J. (2023). Multiple sclerosis: Neuroimmune crosstalk and therapeutic targeting. Cell.

[18] Bettelli E., Oukka M., Kuchroo V.K. (2007). T(H)-17 cells in the circle of immunity and autoimmunity. Nat. Immunol..

[19] Codarri L., Gyulveszi G., Tosevski V., Hesske L., Fontana A., Magnenat L., Suter T., Becher B. (2011). RORgammat drives production of the cytokine GM-CSF in helper T cells, which is essential for the effector phase of autoimmune neuroinflammation. Nat. Immunol..

[20] Lassmann H., van Horssen J., Mahad D. (2012). Progressive multiple sclerosis: Pathology and pathogenesis. Nat. Rev. Neurol..

[21] Ludwin S.K., Rao V., Moore C.S., Antel J.P. (2016). Astrocytes in multiple sclerosis. Mult. Scler..

[22] Buron M.D., Chalmer T.A., Sellebjerg F., Barzinji I., Danny B., Christensen J.R., Christensen M.K., Hansen V., Illes Z., Jensen H.B. (2020). Initial high-efficacy disease-modifying therapy in multiple sclerosis: A nationwide cohort study. Neurology.

[23] van der Star B.J., Vogel D.Y., Kipp M., Puentes F., Baker D., Amor S. (2012). In vitro and in vivo models of multiple sclerosis. CNS Neurol. Disord. Drug Targets.

[24] Pachner A.R. (2011). Experimental models of multiple sclerosis. Curr. Opin. Neurol..

[25] Bittner S., Afzali A.M., Wiendl H., Meuth S.G. (2014). Myelin oligodendrocyte glycoprotein (MOG35-55) induced experimental autoimmune encephalomyelitis (EAE) in C57BL/6 mice. J. Vis. Exp. JoVE.

[26] Bore L., Honda T., Gribble G.W. (2002). Partial synthesis of 23-hydroxyursolic acid isolated from medicinal plants of the Rubiaceae family. Nat. Prod. Lett..

[27] Nguyen H.N., Ahn Y.J., Medina E.A., Asmis R. (2018). Dietary 23-hydroxy ursolic acid protects against atherosclerosis and obesity by preventing dyslipidemia-induced monocyte priming and dysfunction. Atherosclerosis.

[28] Raphael I., Gomez-Rivera F., Raphael R.A., Robinson R.R., Nalawade S., Forsthuber T.G. (2019). TNFR2 limits proinflammatory astrocyte functions during EAE induced by pathogenic DR2b-restricted T cells. JCI Insight.

[29] Bar-Or A., Li R. (2021). Cellular immunology of relapsing multiple sclerosis: Interactions, checks, and balances. Lancet Neurol..

[30] Dong Y., Yong V.W. (2019). When encephalitogenic T cells collaborate with microglia in multiple sclerosis. Nat. Rev. Neurol..

[31] Hohlfeld R., Dornmair K., Meinl E., Wekerle H. (2016). The search for the target antigens of multiple sclerosis, part 1: Autoreactive CD4+ T lymphocytes as pathogenic effectors and therapeutic targets. Lancet Neurol..

[32] Ahn Y.J., Wang L., Foster S., Asmis R. (2020). Dietary 23-hydroxy ursolic acid protects against diet-induced weight gain and hyperglycemia by protecting monocytes and macrophages against nutrient stress-triggered reprogramming and dysfunction and preventing adipose tissue inflammation. J. Nutr. Biochem..

[33] Epstein L.G., Prineas J.W., Raine C.S. (1983). Attachment of myelin to coated pits on macrophages in experimental allergic encephalomyelitis. J. Neurol. Sci..

[34] Bruck W., Sommermeier N., Bergmann M., Zettl U., Goebel H.H., Kretzschmar H.A., Lassmann H. (1996). Macrophages in multiple sclerosis. Immunobiology.

[35] Prineas J.W., Parratt J.D.E. (2021). Multiple Sclerosis: Microglia, Monocytes, and Macrophage-Mediated Demyelination. J. Neuropathol. Exp. Neurol..

[36] Zhang Y., Li X., Ciric B., Curtis M.T., Chen W.J., Rostami A., Zhang G.X. (2020). A dual effect of ursolic acid to the treatment of multiple sclerosis through both immunomodulation and direct remyelination. Proc. Natl. Acad. Sci. USA.

[37] Correale J., Farez M.F. (2015). The Role of Astrocytes in Multiple Sclerosis Progression. Front. Neurol..

[38] Rothhammer V., Quintana F.J. (2015). Control of autoimmune CNS inflammation by astrocytes. Semin. Immunopathol..

[39] Rothhammer V., Kenison J.E., Tjon E., Takenaka M.C., de Lima K.A., Borucki D.M., Chao C.C., Wilz A., Blain M., Healy L. (2017). Sphingosine 1-phosphate receptor modulation suppresses pathogenic astrocyte activation and chronic progressive CNS inflammation. Proc. Natl. Acad. Sci. USA.

[40] Li T., Chen X., Zhang C., Zhang Y., Yao W. (2019). An update on reactive astrocytes in chronic pain. J. Neuroinflamm..

[41] Kim H.S., Tavakoli S., Piefer L.A., Nguyen H.N., Asmis R. (2016). Monocytic MKP-1 is a Sensor of the Metabolic Environment and Regulates Function and Phenotypic Fate of Monocyte-Derived Macrophages in Atherosclerosis. Sci. Rep..

[42] Kim H.S., Ullevig S.L., Zamora D., Lee C.F., Asmis R. (2012). Redox regulation of MAPK phosphatase 1 controls monocyte migration and macrophage recruitment. Proc. Natl. Acad. Sci. USA.

